# Health Tract, No. 287

**Published:** 1867-04

**Authors:** 


### SPRING DANGERS.

Thousands of families are every year bereaved of the light and joy of the household, in the person of the little first-born especially, before the young mother has had experience to guide her in the preservation of the health and lives of children, but it is an easily avoided bereavement. Croup, Diptheria and Putrid Sore Throat have their fruitful cause in allowing little children to become chilled in the harsh, raw, penetrating damp atmosphere of sunset in spring time. Out-door air is certainly beneficial, but not always so. Children under twelve years of age ought to be studiously kept within doors after half an hour before sun-down until the first week in May at least. There is a chilliness in the air about sun-down in Spring which often makes, even grown persons in good health, uncomfortable in mind and body, penetrating and chilling the whole frame, and much more pernicious will it be to the tender constitutions of childhood.

In the afternoons of balmy Spring days children engage in their plays and pastimes out of doors with great energy, the novelty of it inducing them to exercise beyond their strength, and as sun-down approaches, it finds them over-heated, weary in body and with a mind robbed of all its exhilaration; in this condition, the only safety is in bringing them into a warm room the very moment they cease their exercise, for if allowed to stand or sit, or loiter about in the cool of the evening, when more or less of a chill wind is always stirring, it requires but a very few minutes of such exposure to chill the whole body, which, falling on the throat or lungs, may prove fatal in any twenty-four hours.

Another fruitful source of these fatal diseases in children is in allowing them to get their feet wet in stormy, thawy or muddy weather; hence, the moment a child comes in-doors from play in Spring time, the feet should be examined by drawing off shoes and stockings, so as to be able to see and feel if attention is needed.

A third cause of death by the above-named diseases in Spring time is over haste in removing winter clothing; the thickest flannel of mid-winter should be worn by all, without any change to a thinner material, until the middle of May. This applies to old and young, and it is safest not to wear thinner outer clothing, because any given degree of temperature in Spring is really colder by 15 or 20 degrees than in the Fall, because the atmosphere is saturated with dampness which carries the heat from the body with great rapidity; besides this, the changes in Spring are more sudden and violent than in Autumn. There should be a bright, cheerful fire kept burning every day in the family room of every household until the middle of May north of 30 degrees north latitude. This one precaution alone would save multitudes of lives every year. The following tracts are republished to amplify the subject.

